# An Internet-Based Self-Testing Model (Easy Test): Cross-Sectional Survey Targeting Men Who Have Sex With Men Who Never Tested for HIV in 14 Provinces of China

**DOI:** 10.2196/11854

**Published:** 2019-05-15

**Authors:** Xia Jin, Junjie Xu, M Kumi Smith, Dong Xiao, Erica R Rapheal, Xiangfei Xiu, Zhengwei Ding, Yang Zhang, Yang Jie, Ying Liao, Ningxiao Cao, Hao Wu, Yugang Bao

**Affiliations:** 1 AIDS Healthcare Foundation China Program Beijing China; 2 Department of Laboratory Medicine Key Laboratory of AIDS Immunology of National Health and Family Planning Commission The First Affiliated Hospital, China Medical University Shenyang China; 3 School of Public Health University of Minnesota Twin Cities Minneapolis, MN United States; 4 Tongzhi Welfare Beijing China; 5 Chinese Academy of Medical Sciences Hospital for Skin Diseases and STI Control Nanjing China; 6 Beijing Youan hospital Beijing China

**Keywords:** internet, men who have sex with men (MSM), HIV, China

## Abstract

**Background:**

With China’s explosive internet growth, activities such as socializing and partner seeking among men who have sex with men (MSM) has also become Web based through popular services such as Blued. This creates a new mode of health promotion with the potential to instantly reach large numbers of MSM, including those who rarely access traditional offline testing facilities.

**Objective:**

This study aimed to assess the feasibility of the Easy Test in increasing access and uptake of HIV testing and treatment services among MSM and to identify demographic and behavioral predictors of program uptake to inform future implementation.

**Methods:**

A feasibility study of the Easy Test model was conducted from October 2017 to December 2017 in 14 Chinese provinces. Applicants who provided informed consent completed a self-administered questionnaire and submitted a US $5 deposit to have the free test kit delivered to their homes. Orders were then received, processed, and posted by volunteers from local community-based organizations. Once applicants submitted images of their test results, the deposit was refunded to the applicant. Those whose test results were deemed to be HIV-positive were then connected to a peer navigator to accompany the individual to follow-up medical services. A chi-squared trend test was used to assess the relationship between lifetime HIV testing volume and HIV prevalence. Logistic regression models were used to identify independent risk factors associated with two outcomes: (1) never having tested for HIV and (2) receiving an HIV-positive result.

**Results:**

A total of 879 individuals submitted Web-based requests for test kits. Their median age was 28 (interquartile range 24-34 years); 69.3% (609/879) had at least a college education, and 51.5% (453/879) had a monthly income between US $450 to $750; 77.7% (683/879) of the applicants submitted images of their test results, among whom 14.3% (98/683) had an HIV-positive result. Among the 42.9% (293/683) who were first-time testers, the HIV prevalence was 18.8% (55/293). Nearly three-quarters (71/98, 72.4%) of those with a positive test result were connected with a peer navigator and enrolled in treatment. Among the first-time testers, having multiple sexual partners (2-3 sexual partners: adjusted odds ratio [aOR] 2.44, 95% CI 1.08-5.50; 4 or above sexual partners: aOR 3.55, 95% CI 1.18-10.68) and reporting inconsistent condom use in the previous 3 months (aOR 7.95, 95% CI 3.66-17.26) were both associated with an HIV-positive result. An inverse dose response relationship between lifetime HIV testing volume and HIV prevalence was also observed in this study (χ^2^_3_=55.0; *P*<.001).

**Conclusions:**

The Easy Test model reached a larger portion of first-time testers, many who reported higher risk sexual behaviors. This highlights the potential for an internet-based self-test model to increase access to HIV treatment services for HIV-positive MSM in China.

## Introduction

### Background

Men who have sex with men (MSM) represent an increasing proportion of newly reported HIV/AIDS cases. In 2017, MSM accounted for 25.54% (34,358/134,512) of all newly reported HIV/AIDS cases in China, compared with 14.70% (10,954/74,517) in 2011 [1,2]. In addition, national sentinel surveillance data indicate an increase in HIV prevalence among MSM from 5.72% (1948/34,009) to 7.98% (3312/41,503) between 2010 and 2015 [3]. Although the Chinese government has provided free HIV testing and counseling since 2003, and health authorities have affected a massive scale-up of facility-based HIV testing services, experts estimate around half of Chinese MSM remain untested for HIV [4].

Facility-based HIV testing is defined as HIV testing conducted in health care facilities, including the Centers for Disease Control and Prevention (CDC), hospitals and clinics, and community-based organizations (CBOs) that conduct HIV antibody screening (often using rapid tests) with linkage services to health care facilities for those who screen positive. The public health utility of internet-based interventions is of growing interest in China owing to the expansion of internet access—internet and mobile internet users currently exceed 800 million people [5]—and the growing use of social networking apps for socializing and sexual partner seeking [6-9]. To date, internet-based interventions have been used to promote HIV testing in young MSM, promote HIV/hepatitis C virus testing among people who inject drugs, reduce HIV transmission among male sex workers, reduce pretreatment loss to follow-up in HIV-diagnosed individuals, and improve the adherence of antiretroviral therapy in people living with HIV/AIDS [10-14]. Although 73.49% (34,712/47,231) of respondents in a nationally representative survey of MSM reported that they had found recent sex partners on the Web, only 26.36% (3890/14,757) of these respondents indicated that they had been tested for HIV and received test results in the last year [15,16]. The high proportion of Chinese MSM who use the internet to find sexual partners and the low rate of HIV testing among internet-using MSM underscore the potential of internet-based interventions to increase HIV testing uptake in this population.

The World Health Organization has recently recommended HIV self-testing to increase HIV testing uptake in light of its convenience, acceptability, timeliness, accuracy, and its provision of privacy to testers [17]. The Chinese Center for Disease Control and Prevention (China CDC) has also cited self-testing as a priority area. However, public health concerns about self-testing, including the risk of the clients misinterpreting results and missed opportunities for safe sex counseling and linkage to care, have slowed its implementation beyond small-scale studies in urban areas [6,18]. To address these shortcomings, the AIDS Healthcare Foundation (AHF) China developed an internet-based self-testing model, Easy Test, in which clients were invited to place Web-based orders for home delivery of free HIV self-test kits. For those testing positive, access to other services (eg, test result confirmation by trained staff or linkage to an in-person peer navigator) would be provided as well. The internet-based approach of Easy Test was designed with the explicit goal of increasing test coverage among MSM, particularly those who may be less likely to access facility-based HIV testing and treatment services.

### Objectives

The objectives of this study were (1) to assess the capability of the Easy Test model in increasing access to HIV testing and treatment services for MSM who have never tested for HIV and (2) to identify demographic and behavioral predictors of program uptake to inform future implementation.

## Methods

### Study Setting and Sampling Strategy

This study was conducted by AHF China from October 2017 to December 2017 in 14 Chinese provinces. The Easy Test model was born out of a collaborative effort between AHF China, regional health facilities, and MSM CBOs local to each of the study cities (Beijing, Tianjin, Shanxi, Chongqing, Liaoning, Guangxi, Yunnan, Hunan, Hainan, Sichuan, Henan, Heilongjiang, Zhejiang, and Xinjiang). Promotion of the Easy Test program was carried out using social media tools (WeChat, QQ, and Blued) as well as offline events organized by local CBOs. Printed Easy Test promotion cards were also included in the self-testing kit packages to encourage participants to share information about the service with spouses, sexual partners, or friends. Eligible participants were at least aged 16 years, born biologically male, had engaged in anal sex with a man, and were of negative or unknown HIV status.

### Study Procedures

Participants interested in ordering a self-test kit were instructed to visit the Easy Test website either by typing in the URL or scanning an exclusive Quick Response code. Following eligibility screening, those meeting the requirements were asked to provide electronic informed consent and to participate in a self-administered survey where they provided information on sociodemographic characteristics, sexual risk behaviors, and HIV test history. Participants were also asked for their mobile phone number, shipping address, a US $5 deposit, and a nonrefundable shipping fee of US $2 to $3. All payments were processed using the secure and widely used Web-based payment platforms of Alipay or WeChat. Peer educators at the MSM CBOs processed all incoming orders and dispatched all express mail deliveries with China’s most widely used express delivery service. Packages were delivered within 1 to 3 days in an unmarked package with no visible indications of the contents or sender. Self-testing kits were limited to 1 per registered mobile phone number to minimize potential bias from repeat testers.

The self-testing package included a rapid test reagent, a single-use safety lancet, a buffer, a dropper, an alcohol pad, a printed Easy Test promotion card, and printed kit instructions. The rapid test reagent used in this study is Alere Determine TM HIV-1/2 (Alere Medical). The needle of the single use safety spring lancet is designed to permanently and automatically retract after the first use. Participants were also offered to be put in touch with a peer educator, who could provide over-the-phone guidance on self-administration of the test. In addition, an instructional video was provided on the Easy Test website and WeChat account. Participants were refunded their US $5 deposit upon successful upload of a photograph of the results of their completed self-test kit, which was then interpreted by trained staff at each CBO and health facility.

Volunteers from each local CBO contacted participants whose submitted photographs indicated an HIV-positive result to arrange linkage services. Those interested in linkage services were paired with a peer navigator to accompany them to receive confirmatory testing, as well as initial visits for treatment and care following formal diagnosis. Treatment and care for HIV/AIDS are available free through the Chinese national treatment program [19]. The observation period to assess the rate of HIV treatment initiation among diagnosed individuals in this study extended a month beyond the study endpoint to maximize inclusion of successful referrals. Peer navigators were trained volunteers from local CBOs who paired up with local health departments to help ensure successful linkage of participants to confirmatory testing, care, and treatment.

### Data Analysis

The information on sociodemographic characteristics, sexual behaviors, and HIV testing history collected as part of the self-testing application was used for descriptive analyses. A chi-squared trend test was used to assess the relationship between lifetime HIV testing volume and HIV prevalence. Independent risk factors for never having tested for HIV and a positive self-test result were assessed using bivariable logistic regression analysis. Variables significant at *P* ≤.05 or determined to be clinically relevant were selected for inclusion in the multivariable logistic model. All statistical analyses were performed using SAS 9.4 (SAS Institute Inc).

### Ethical Statement

The study received approval from the Hospital for Skin Diseases and STI Control of Chinese Academy of Medical Sciences.

## Results

### Study Participants

**Figure 1:**
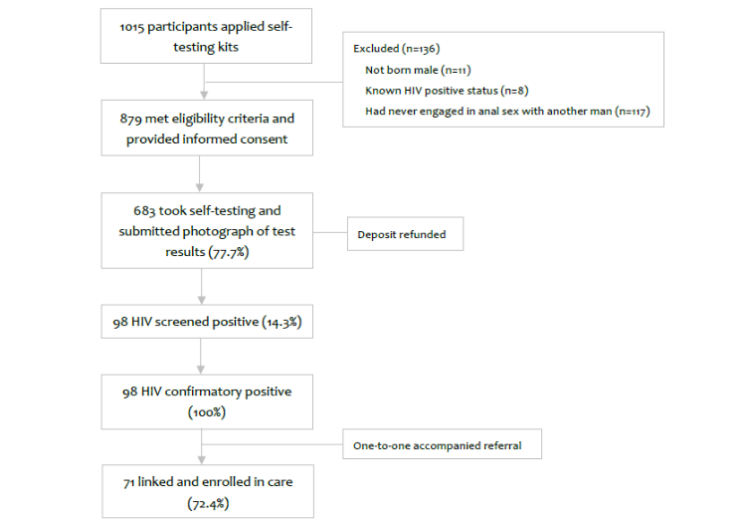
Flowchart of the Easy Test model.

From October 2016 to December 2016, a total of 1015 participants applied for Web-based self-testing kits among whom 879 (86.60%) met the eligibility criteria and provided informed consent. Those who did not meet the requirements included 11 individuals who reported their sex at birth as female, 8 who were already aware of their HIV-positive status, and 117 who had never engaged in anal sex with a man (Figure 1).

### Sociodemographics and Behaviors

Among the 879 eligible participants, the median age was 28 years (interquartile range [IQR] 24-34 years), 91.9% (808/879) were of Han ethnicity, 69.3% (609/879) had a college education or higher, 74.3% (653/879) had never been married, 12.7% (112/879) were currently students, and half (453/879, 51.5%) had a monthly income between 3000 and 5000 Chinese yuan (US $450 to $750).

Overall, 90.0% (791/879) of participants self-identified as homosexual and the median age of sexual debut among the participants was 20 years (IQR 18-22 years). Among the 87.1% (766/879) who had engaged in anal sex with another man in the last 3 months, 53.3% (408/766) had had 2 or more partners and 28.9% (221/766) had not used condoms during anal intercourse. Among all participants, 81.5% (716/879) reported condom use during their last anal intercourse, although only 60.5% (433/716) reported correct condom use; 10.0% (88/879) of participants self-identified as bisexual or unsure, 96.3% (70/88) reported vaginal sex with a female partner, and only 31.4% (22/70) of these reported condom use during vaginal sex in the last 3 months; 29.4% (258/879) of participants reported that they were aware of the HIV status of regular sexual partners.

Participants who self-reported incorrect condom use (adjusted odds ratio [aOR] 1.63, 95% CI 1.17-2.27) and lack of knowledge about their regular sexual partners’ HIV status (aOR 2.45, 95% CI 1.67-3.60) were associated with having never tested for HIV before participation in this study compared with participants reporting correct condom use and awareness of the HIV status of sexual partners. Further details are provided in Table 1.

### Testing History, Results, and Linkage to Care

Upon enrollment in this study, 40.0% (352/879) of the participants reported having never tested for HIV before. Over three-quarters (683/879, 77.7%) of participants submitted a photograph of the completed self-test result to the study staff. The overall HIV prevalence among these participants was 14.3% (98/683), among whom 100% were eventually confirmed as HIV infection following confirmatory testing at a local health facility. Participants with confirmed HIV were all peer-navigated to local health facilities and 72% (71/98) of them enrolled in treatment with a 1-month extension beyond the study endpoint.

**Figure 2:**
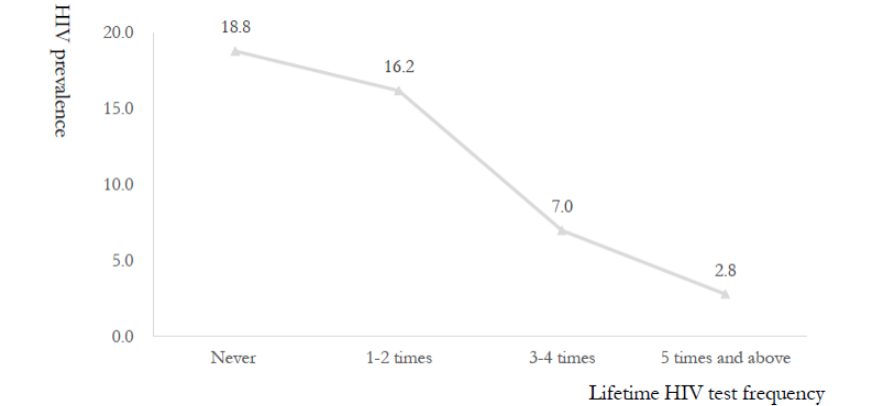
HIV prevalence with lifetime frequency.

Nearly half of the participants (293/683, 42.9%) who submitted photographs of their self-test results were first-time testers for HIV, and among whom HIV prevalence was 18.8% (55/293). Among those who had tested before, we observed an inverse dose response relationship between lifetime HIV testing volume and HIV prevalence. In the subgroups of participants who had tested once or twice previously versus 3 to 4 times versus 5 or more times, HIV prevalence was 16.2% (33/204), 7.0% (8/114), and 2.8% (2/72), respectively (χ^2^_3_=55.0; *P*<.001; Figure 2).

**Table 1:** Demographics and behaviors of the Easy Test participants and factors associated with those who had never tested for HIV.

Variable		Participants, n (%)	Never tested for HIV, n (%)	Ever tested for HIV, n (%)	Unadjusted OR^a^ (95% CI)	Adjusted OR (95% CI)	*P* value for adjusted OR
**Age (years)**							
	<25	234 (26.6)	118 (33.5)	116 (22.0)	2.26 (1.53-3.35)	2.08 (1.25-3.44)	.005
	25-34	442 (50.3)	171 (48.6)	271 (51.4)	1.40 (0.98-2.00)	1.78 (1.15-2.75)	.01
	≥35	203 (23.1)	63 (17.9)	140 (26.6)	1.0	1.0	N/A^b^
**Ethnicity**							
	Han	808 (91.9)	336 (95.5)	472 (89.6)	2.45 (1.38-4.34)	3.49 (1.62-7.52)	.001
	Other	71 (8.1)	16 (4.5)	55 (10.4)	1.0	1.0	N/A
**Education**							
	<12 years	270 (30.7)	122 (34.7)	148 (28.1)	1.36 (1.02-1.82)	N/A	N/A
	≥12 years	609 (69.3)	230 (65.3)	379 (71.9)	1.0	N/A	N/A
**Marital status**							
	Never married	653 (74.3)	269 (76.4)	384 (72.9)	1.21 (0.88-1.65)	N/A	N/A
	Other^c^	226 (25.7)	83 (23.6)	143 (27.1)	1.0	N/A	N/A
**Occupation**							
	Students	112 (12.7)	60 (17.0)	52 (9.9)	1.88 (1.26-2.80)	N/A	N/A
	Other	767 (87.3)	292 (83.0)	475 (90.1)	1.0	N/A	N/A
**Monthly income (US $)**							
	<450	194 (22.1)	107 (30.4)	87 (16.5)	3.30 (2.20-4.95)	2.29 (1.37-3.84)	.002
	450-749	453 (51.5)	182 (51.7)	271 (51.4)	1.80 (1.28-2.54)	1.31 (0.86-1.98)	>.05
	≥750	232 (26.4)	63 (17.9)	169 (32.1)	1.0	1.0	N/A
**Age of sexual debut (years)**							
	<18	142 (16.2)	54 (15.3)	88 (16.7)	0.90 (0.63-1.31)	N/A	N/A
	≥18	737 (83.8)	298 (84.7)	439 (83.3)	1.0	N/A	N/A
**Sexual orientation^d^**							
	Homosexual	791 (90.0)	325 (92.3)	466 (88.4)	1.58 (0.98-2.53)	N/A	N/A
	Bisexual/Unsure	88 (10.0)	27 (7.7)	61 (11.6)	1.0	N/A	N/A
**Sexual role**							
	Insertive partner	295 (33.6)	107 (30.4)	188 (35.7)	1.0	N/A	N/A
	Both	332 (37.8)	128 (36.4)	204 (38.7)	1.10 (0.80-1.53)	N/A	N/A
	Receptive partner	252 (28.6)	117 (33.2)	135 (25.6)	1.52 (1.08-2.15)	N/A	N/A
**Number of anal sexual partners in the last 3 months**							
	1	358 (46.7)	150 (50.8)	208 (44.2)	1.0	N/A	N/A
	2-3	335 (43.7)	113 (38.4)	222 (47.1)	0.71 (0.52-0.96)	N/A	N/A
	≥4	73 (9.6)	32 (10.8)	41 (8.7)	1.08 (0.65-1.80)	N/A	N/A
**Always used condoms during anal sex in the last 3 months**							
	Yes	545 (71.1)	187 (63.4)	358 (76.0)	1.0	N/A	N/A
	No	221 (28.9)	108 (36.6)	113 (24.0)	1.83 (1.33-2.51)	N/A	N/A
**Correct condom use during last anal sex^e^**							
	Yes	433 (60.5)	139 (52.3)	294 (65.3)	1.0	1.0	N/A
	No	283 (39.5)	127 (47.7)	156 (34.7)	1.72 (1.26-2.35)	1.63 (1.17-2.27)	.004
**Know the HIV status of regular sexual partners**							
	Yes	258 (29.4)	62 (17.6)	196 (37.2)	1.0	1.0	N/A
	No	621 (70.6)	290 (82.4)	331 (62.8)	2.77 (2.00-3.84)	2.45 (1.67-3.60)	<.001

^a^OR: odds ratio

^b^N/A: not applicable.

^c^Marital status: “other” included those who were married, cohabitating, divorced, or widowed.

^d^Sexual orientation is not a behavior but an identity.

^e^Correct condom use was defined as proper use of a condom during the entire process of sexual intercourse (from the beginning of anal sex to the end) without breakage or falling off.

### Factors Associated With HIV Infection

Results of multivariate models indicated that overall, participants who reported usually being the receptive anal sex partner (aOR 4.58, 95% CI 1.52-13.79) or who assumed both insertive and receptive roles (aOR 4.10, 95% CI 1.39-12.12) had far greater odds of HIV infection relative to those who mainly assumed the insertive role. Participants reporting greater numbers of sexual partners in the last 3 months also had higher odds of HIV infection, with an aOR of 2.74 (95% CI 1.23-6.11) for those with 2 to 3 sexual partners and 3.98 (95% CI 1.27-12.49) for 4 or more sexual partners, compared with those reporting only 1 partner in the same time period. The odds of HIV infection were also higher for those reporting inconsistent condom use during anal intercourse in the last 3 months (aOR 4.64, 95% CI 2.23-9.63) and those reporting incorrect condom use during their most recent anal sex episodes had higher odds of HIV infection (aOR 2.19, 95% CI 1.05-4.58) compared with those reporting consistent and correct usage. Other characteristics associated with higher odds of HIV infection included fewer than 3 lifetime HIV testing episodes (aOR 2.58, 95% CI 1.02-6.49), no knowledge of sexual partners’ HIV infection status (aOR 3.26, 95% CI 1.06-10.00), and never having been married (aOR 4.10, 95% CI 1.31-12.82).

Among the subset of participants who had never tested for HIV, those who reported being a receptive anal partner or who assumed both insertive and receptive roles had far higher odds of HIV infection (aOR 3.78, 95% CI 1.38-10.35 for receptive partners and aOR 3.00, 95% CI 1.09-8.26 for both positions) relative to those who mainly assumed the insertive role. Participants reporting greater numbers of sexual partners in the last 3 months also had higher odds of HIV infection, with an aOR of 2.44 (95% CI 1.08-5.50) for those with 2 to 3 sexual partners and 3.55 (95% CI 1.18-10.68) for 4 or more sexual partners, compared with those reporting only 1 partner in the same period. The likelihood of HIV infection was higher among participants who did not always use condoms during anal intercourse in the last 3 months (aOR 7.95, 95% CI 3.66-17.26) relative to participants who had used condoms during anal sex in the last 3 months. The details are provided in Tables 2 and 3.

**Table 2:** Factors correlated with HIV infection among the Easy Test participants in China.

Characteristics		Participants (N=879), n	HIV cases, n	Prevalence, %	Unadjusted OR^a^ (95% CI)	Adjusted OR (95% CI)	Adjusted *P* value
**Age (years)**							
	<25	193	35	18.1	1.76 (0.95-3.25)	N/A^b^	N/A
	25-34	329	45	13.7	1.26 (0.70-2.25)	N/A	N/A
	≥35	161	18	11.2	1.0	N/A	N/A
**Ethnicity**							
	Han	624	93	14.9	1.89 (0.74-4.85)	N/A	N/A
	Other	59	5	8.5	1.0	N/A	N/A
**Education**							
	<12 years	227	29	12.8	0.82 (0.52-1.31)	N/A	N/A
	≥12 years	456	69	15.1	1.0	N/A	N/A
**Marital status**							
	Never married	499	83	16.6	2.25 (1.26-4.01)	4.10 (1.31-12.82)	.015
	Other^c^	184	15	8.2	1.0	1.0	N/A
**Occupation**							
	Students	91	17	18.7	1.45 (0.81-2.58)	N/A	N/A
	Other	592	81	13.7	1.0	N/A	N/A
**Monthly income (US $)**							
	<450	155	25	16.1	2.40 (1.13-5.06)	N/A	N/A
	450-749	380	62	16.3	2.43 (1.24-4.75)	N/A	N/A
	≥750	148	11	7.4	1.0	N/A	N/A
**Age of sexual debut (years)**							
	<18	128	21	16.4	1.22 (0.72-2.06)	N/A	N/A
	≥18	555	77	13.9	1.0	N/A	N/A
**Sexual orientation^d^**							
	Homosexual	615	93	15.1	2.25 (0.88-5.73)	N/A	N/A
	Bisexual/Unsure	68	5	7.4	1.0	N/A	N/A
**Sex role**							
	Insertive partner	230	14	6.1	1.0	1.0	N/A
	Both	246	43	17.5	3.27 (1.74-6.15)	4.10 (1.39-12.12)	.011
	Receptive partner	207	41	19.8	3.81 (2.01-7.22)	4.58(1.52-13.79)	.007
**Number of anal sexual partners in the last 3 months**							
	1	272	27	9.9	1.0	1.0	N/A
	2-3	246	52	21.1	2.43 (1.47-4.02)	2.74 (1.23-6.11)	.014
	≥4	64	13	20.3	2.31 (1.12-4.79)	3.98 (1.27-12.49)	.018
**Always used condoms during anal sex in the last 3 months**							
	Yes	393	26	6.6	1.0	1.0	N/A
	No	189	66	34.9	7.57 (4.61-12.46)	4.64 (2.23-9.63)	<.001
**Correct condom use during last anal sex^e^**							
	Yes	298	17	5.7	1.0	1.0	N/A
	No	248	39	15.7	3.08 (1.70-5.60)	2.19 (1.05-4.58)	.037
**Lifetime HIV testing frequency**							
	<3	497	88	16.2	3.79 (1.92-7.46)	2.58 (1.02-6.49)	.045
	≥3	10	186	2.8	1.0	1.0	N/A
**Know the HIV status of regular sexual partners**							
	Yes	157	10	6.4	1.0	1.0	N/A
	No	526	88	16.7	2.95 (1.50-5.83)	3.26 (1.06-10.00)	.04

^a^OR: odds ratio.

^b^N/A: not applicable.

^c^Marital status: “other” included those who were married, cohabitating, divorced, or widowed.

^d^Sexual orientation is not a behavior but an identity.

^e^Correct condom use was defined as proper use of a condom during the entire process of sexual intercourse (from the beginning of anal sex to the end) without breakage and falling off.

**Table 3:** Factors correlated with HIV infection among first-time HIV testers in China.

Characteristics		Participants (N=293), n	HIV cases, n	Prevalence, %	Unadjusted OR^a^ (95% CI)	Adjusted OR (95% CI)	Adjusted *P* value
**Age (years)**							
	<25	98	22	22.4	1.30 (0.57-2.30)	N/A^b^	N/A
	25-34	140	23	16.4	0.89 (0.39-2.01)	N/A	N/A
	≥35	55	10	18.2	1.0	N/A	N/A
**Ethnicity**							
	Han	281	53	18.9	1.16 (0.25-5.46)	N/A	N/A
	Other	12	2	16.7	1.0	N/A	N/A
**Education**							
	<12 years	109	18	16.5	0.79 (0.42-1.46)	0.41 (0.19-0.89)	.025
	≥12 years	184	37	20.1	1.0	1.0	N/A
**Marital status**							
	Never married	224	48	21.4	2.42 (1.04-5.62)	N/A	N/A
	Other^a^	69	7	10.1	1.0	N/A	N/A
**Occupation**							
	Students	49	11	22.4	1.32 (0.62-2.78)	N/A	N/A
	Other^c^	244	44	18	1.0	N/A	N/A
**Monthly income (US $)**							
	<450	88	15	17	1.73 (0.59-5.09)	N/A	N/A
	450-749	158	35	22.2	2.39 (0.88-6.50)	N/A	N/A
	≥750	47	5	10.6	1.0	N/A	N/A
**Age of sexual debut (years)**							
	<18	47	13	27.7	1.86 (0.90-3.82)	N/A	N/A
	≥18	246	42	17.1	1.0	N/A	N/A
**Sexual orientation^d^**							
	Homosexual	273	53	19.4	2.17 (0.49-9.63)	N/A	N/A
	Bisexual/unsure	20	2	10	1.0	N/A	N/A
**Sex role**							
	Insertive partner	94	7	7.4	1.0	1.0	N/A
	Both	97	21	21.6	3.43 (1.38-8.52)	3.00 (1.09-8.26)	.034
	Receptive partner	102	27	26.5	4.47 (1.84-10.86)	3.78 (1.38-10.35)	.01
**Number of anal sexual partners in the last 3 months**							
	1	127	15	11.8	1.0	1.0	N/A
	2-3	87	27	31	3.36 (1.66-6.80)	2.44 (1.08-5.50)	.033
	≥4	26	10	38.5	4.67 (1.79-12.14)	3.55 (1.18-10.68)	.024
**Always used condoms during anal sex in the last 3 months**							
	Yes	148	12	8.1	1.0	1.0	N/A
	No	92	40	43.5	8.72 (4.24-17.91)	7.95 (3.66-17.26)	<.001
**Correct condom use during last anal sex^e^**							
	Yes	104	9	8.7	1.0	N/A	N/A
	No	117	17	14.5	1.79 (0.76-4.22)	N/A	N/A
**Know the HIV status of regular sexual partners**							
	Yes	46	4	8.7	1.0	N/A	N/A
	No	247	51	20.6	2.73 (0.94-7.97)	N/A	N/A

^a^OR: odds ratio.

^b^N/A: not applicable.

^c^Marital status: “other” included those who were married, cohabitating, divorced, or widowed.

^d^Sexual orientation is not a behavior but an identity.

^e^Correct condom use was defined as proper use of a condom during the entire process of sexual intercourse (from the beginning of anal sex to the end) without breakage and falling off.

## Discussion

### Principal Findings

The large sample size, high participation among first-time testers (293/683, 42.9%), and the high rate of photograph-confirmed test completion (683/879, 77.7%) all emphasize the feasibility and efficacy of our Easy Test model for promoting internet-based HIV self-testing and linkage to care among MSM in China. Our study assessed the utility of the Easy Test model as a supplement to existing facility-based HIV testing services. The proportion of those who never tested for HIV in this study (293/683, 42.9%) was higher than in a previous Web-based distribution of self-testing kits in MSM in Guangdong (118/380, 31.1%) [18], though the vastly different regional scope (one province in Zhong et al vs 14 provinces in this study), sample size (N=198 in Zhong et al vs N=1015 in this study), and HIV prevalence (4.5% in Zhong et al vs 18.8% in this study) suggest substantial differences in sample makeup that may impede direct comparison.

All participants who shared their positive test results with study staff were eventually confirmed as HIV infected, an encouraging finding for which we believe there are possible explanations. First, inclusion of a one-to-one peer navigation service for those testing positive may have helped assuage known concerns in the study population regarding what to do in the event of a positive test result. The other reason that contributed to this was the rapid test substitute strategy issued by CDC China in 2015 to reduce the time from screening positive to diagnosis as HIV infected within 1 to 3 days [20]. A similar 100% confirmatory testing result of the screened HIV-positive cases was found in a smaller size study (N=198; 8 positives) in Guangdong [18].

Treatment enrollment rates among those who received confirmatory testing were high (71/98, 72%) but still well below the Joint United Nations Programme on HIV/AIDS target of 90% of diagnosed individuals on therapy. As this proportion captures treatment initiated within a month after study close out, this rate may underestimate the ultimate proportion. However, China’s universal and immediate treatment policies suggest that full linkage may be attainable, and future rollout may do well to consider additional strategies such as recruitment of test coordinators from health facilities to conduct community outreach for diagnosed individuals.

Participants in this study reported more sexual risk behaviors than in comparable Web-based samples of MSM in China, including more multiple sexual partnerships, infrequent or incorrect condom use, more frequent receptive anal sex, and less awareness of sexual partners’ HIV status [6,21]. The overall HIV prevalence (98/683, 14.3%) was also far higher than in a similar sample (24/341, 7.0%) [21]. However, the identified risk factors for HIV infection were similar to these studies and included characteristics such as being the receptive anal sex partner (whether primarily or occasionally so), higher numbers of anal sexual partners, and not always using condoms during anal intercourse [6,22-25]. Even though most of the internet-based MSM in the study reported using condoms during anal sex, there is still a significant proportion who reported inconsistent and incorrect condom use, particularly among those who had previously never tested for HIV (283/716, 39.5%). Our findings suggest that public health officials should pay attention to relevant contextual factors that may increase the risk of HIV infection, including greater numbers of anal sexual partners and unprotected anal sexual activities. In addition, HIV/AIDS health education and condoms should be provided with an emphasis on MSM who prefer the receptive anal role to increase condom use and negotiation skills with their sexual partners [6]. Furthermore, the proportion of HIV-positive results decreased with the increased lifetime HIV testing frequency in this study. This information further highlights the importance of routine HIV testing and its impact on better health outcomes.

### Limitations

The study findings should be considered in light of several limitations. First, our online recruitment methods may have resulted in a sample which over-represents better-educated, literate, and higher income MSM, a well-documented phenomenon of Web-based samples [26-28]. Second, to understand the potential bias introduced by the nonrandom subset of participants who submitted photographs of their self-testing results, we compared reported behaviors across groups among those who did and did not submit photographs of their completed self-test results. This analysis found that condom use during the last anal sex (68.8% for correct condom use vs 87.3% for not) and HIV status of sexual partners (60.9% for knowing partner status vs 84.7% for not) were independent risk factors associated with not providing feedback regarding their results. It is possible that those with lower HIV risk were more reluctant to submit their test results, which could lead to overestimation of HIV prevalence in this population. Finally, we do not know the proportion of respondents with HIV-positive test results who may have sought care and treatment on their own without the help of peer-navigator services provided as a part of this study.

### Conclusions

Our study demonstrated that internet-based self-testing may be an effective approach for increasing HIV test uptake, particularly among those who have never previously tested for HIV. It also shows promise as a strategy for improving linkage to care and treatment initiation among those diagnosed with HIV. Internet-based MSM who engage in self-testing and who have never previously tested for HIV are less likely to use condoms during sex with both male and female partners, suggesting that internet-based testing interventions may be effective in reaching those in greatest need of these services.

## Abbreviations

AHF: AIDS Healthcare Foundation
aOR: adjusted odds ratio
CBO: community-based organization
CDC: Centers for Disease Control and Prevention
IQR: interquartile range
MSM: men who have sex with men

## References

[1] National Center for AIDS/STD Control and Prevention, China CDC (2012). Update on the AIDS/STD epidemic in China and main response in control and prevention in 2011. Chinese Journal of AIDS & STD.

[2] NCAIDS, NCSTD, China CDC (2018). Update on the AIDS/STD epidemic in China in December 2017. Chinese Journal of AIDS & STD.

[3] Ge L, Li D, Li P, Guo W, Cui Y (2017). Population specific sentinel surveillance for HIV infection, syphilis and HCV infection in China, during 2010-2015. Disease Surveillance.

[4] Chow EPF, Wilson DP, Zhang L (2012). The rate of HIV testing is increasing among men who have sex with men in China. HIV Med.

[5] (2018). China Internet Network Information Center (CNNIC).

[6] Pan S, Xu J, Han X, Zhang J, Hu Q, Chu Z, Hai Y, Mao X, Yu Y, Geng W, Jiang Y, Shang H (2016). nternet-based sex-seeking behavior promotes HIV infection risk: a 6-year serial cross-sectional survey to MSM in Shenyang, China. Biomed Res Int.

[7] Tang W, Best J, Zhang Y, Liu F, Tso LS, Huang S, Yang B, Wei C, Tucker JD (2016). Gay mobile apps and the evolving virtual risk environment: a cross-sectional online survey among men who have sex with men in China. Sex Transm Infect.

[8] Zou H, Fan S (2017). Characteristics of men who have sex with men who use smartphone geosocial networking applications and implications for HIV interventions: a systematic review and meta-analysis. Arch Sex Behav.

[9] Grosskopf NA, LeVasseur MT, Glaser DB (2014). Use of the Internet and mobile-based "apps" for sex-seeking among men who have sex with men in New York City. Am J Mens Health.

[10] Bauermeister JA, Pingel ES, Jadwin-Cakmak L, Harper GW, Horvath K, Weiss G, Dittus P (2015). Acceptability and preliminary efficacy of a tailored online HIV/STI testing intervention for young men who have sex with men: the Get Connected! program. AIDS Behav.

[11] Aronson ID, Bennett A, Marsch LA, Bania TC (2017). Mobile technology to increase HIV/HCV testing and overdose prevention/response among people who inject drugs. Front Public Health.

[12] Mimiaga MJ, Thomas B, Biello K, Johnson BE, Swaminathan S, Navakodi P, Balaguru S, Dhanalakshmi A, Closson EF, Menon S, O'Cleirigh C, Mayer KH, Safren SA (2017). A pilot randomized controlled trial of an integrated in-person and mobile phone delivered counseling and text messaging intervention to reduce HIV transmission risk among male sex workers in Chennai, India. AIDS Behav.

[13] Mehta K, Kumar AM, Chawla S, Chavda P, Selvaraj K, Shringarpure KS, Solanki DM, Verma PB, Rewari BB (2018). 'M-TRACK' (mobile phone reminders and electronic tracking tool) cuts the risk of pre-treatment loss to follow-up by 80% among people living with HIV under programme settings: a mixed-methods study from Gujarat, India. Glob Health Action.

[14] Kanters S, Park JJ, Chan K, Socias ME, Ford N, Forrest JI, Thorlund K, Nachega JB, Mills EJ (2017). Interventions to improve adherence to antiretroviral therapy: a systematic review and network meta-analysis. Lancet HIV.

[15] Wu Z, Xu J, Liu E, Mao Y, Xiao Y, Sun X, Liu Y, Jiang Y, McGoogan JM, Dou Z, Mi G, Wang N, Sun J, Liu Z, Wang L, Rou K, Pang L, Xing W, Xu J, Wang S, Cui Y, Li Z, Bulterys M, Lin W, Zhao J, Yip R, Wu Y, Hao Y, Wang Y, National MSM Survey Group (2013). HIV and syphilis prevalence among men who have sex with men: a cross-sectional survey of 61 cities in China. Clin Infect Dis.

[16] Liu M, Ma B, Mi G (2016). A comparison of web-based survey national sentinel surveillance among MSM in China. Chin J AIDS STD.

[17] (2016). World Health Organization.

[18] Zhong F, Tang W, Cheng W, Lin P, Wu Q, Cai Y, Tang S, Fan L, Zhao Y, Chen X, Mao J, Meng G, Tucker JD, Xu H (2017). Acceptability and feasibility of a social entrepreneurship testing model to promote HIV self-testing and linkage to care among men who have sex with men. HIV Med.

[19] Hao Y, Sun X, Xia G, Jiao Y, Song L, Shen J, Mao Y, Zhang F, Jiang Y, Rou K, Wang W, Zhao Y, Jiao Z, Liu Q, Yi L, Shi Y, Wang A, Lv F, Wang L, Liu Y, Wang X, Cui Y, Jin C, Wu D, Hu H, Chen Q, Zhang X, Wang N, Liu Z, Sun J, Liu K, Han M, Wu Z (2014). Program in HIV/AIDS prevention and treatment since implementing the Four Frees and One Care AIDS policy in China. Chin J AIDS STD.

[20] (2015). National Guideline for Detection of HIV/AIDS.

[21] Qin Y, Liu F, Tang W, Tang S, Liu C, Mao J, Wei C, Tucker J (2016). HIV self-testing among high-risk men who have sex with men in China: a cross-sectional study. Lancet.

[22] Xu J, An M, Han X, Jia M, Ma Y, Zhang M, Hu Q, Chu Z, Zhang J, Jiang Y, Geng W, Lu L, Shang H (2013). Prospective cohort study of HIV incidence and molecular characteristics of HIV among men who have sex with men(MSM) in Yunnan Province, China. BMC Infect Dis.

[23] Zhang W, Xu J, Zou H, Zhang J, Wang N, Shang H (2016). HIV incidence and associated risk factors in men who have sex with men in Mainland China: an updated systematic review and meta-analysis. Sex Health.

[24] Xu J, Zhang C, Hu Q, Chu Z, Zhang J, Li Y, Lu L, Wang Z, Fu J, Chen X, Yan H, Zhuang M, Jiang Y, Geng W, Vermund SH, Shang H, Qian H (2014). Recreational drug use and risks of HIV and sexually transmitted infections among Chinese men who have sex with men: Mediation through multiple sexual partnerships. BMC Infect Dis.

[25] Xu J, Zhang M, Brown K, Reilly K, Wang H, Hu Q, Ding H, Chu Z, Bice T, Shang H (2010). Syphilis and HIV seroconversion among a 12-month prospective cohort of men who have sex with men in Shenyang, China. Sex Transm Dis.

[26] Ross MW, Tikkanen R, Månsson SA (2000). Differences between internet samples and conventional samples of men who have sex with men: implications for research and HIV interventions. Soc Sci Med.

[27] Ross MW, Månsson SA, Daneback K, Cooper A, Tikkanen R (2005). Biases in internet sexual health samples: comparison of an internet sexuality survey and a national sexual health survey in Sweden. Soc Sci Med.

[28] Wang C, Mollan KR, Hudgens MG, Tucker JD, Zheng H, Tang W, Ling L (2018). Generalisability of an online randomised controlled trial: an empirical analysis. J Epidemiol Community Health.

